# Superoxide Dismutase 1 Loss Disturbs Intracellular Redox Signaling, Resulting in Global Age-Related Pathological Changes

**DOI:** 10.1155/2014/140165

**Published:** 2014-09-08

**Authors:** Kenji Watanabe, Shuichi Shibuya, Yusuke Ozawa, Hidetoshi Nojiri, Naotaka Izuo, Koutaro Yokote, Takahiko Shimizu

**Affiliations:** ^1^Department of Advanced Aging Medicine, Chiba University Graduate School of Medicine, Chiba 260-8670, Japan; ^2^Department of Clinical Cell Biology and Medicine, Chiba University Graduate School of Medicine, Chiba 260-8670, Japan; ^3^Department of Orthopaedics, Juntendo University Graduate School of Medicine, Bunkyo-ku, Tokyo 113-0033, Japan

## Abstract

Aging is characterized by increased oxidative stress, chronic inflammation, and organ dysfunction, which occur in a progressive and irreversible manner. Superoxide dismutase (SOD) serves as a major antioxidant and neutralizes superoxide radicals throughout the body. *In vivo* studies have demonstrated that copper/zinc superoxide dismutase-deficient (*Sod1^−/−^*) mice show various aging-like pathologies, accompanied by augmentation of oxidative damage in organs. We found that antioxidant treatment significantly attenuated the age-related tissue changes and oxidative damage-associated p53 upregulation in *Sod1^−/−^* mice. This review will focus on various age-related pathologies caused by the loss of *Sod1* and will discuss the molecular mechanisms underlying the pathogenesis in *Sod1^−/−^* mice.

## 1. Introduction

Aging is associated with several functional and structural deficits in organs, which are linked to biochemical changes, including oxidative modifications, protein aggregation, and altered gene expression [1]. Reactive oxygen species (ROS) are mainly generated from the electron transport chain in mitochondria and nonspecifically oxidize cellular molecules such as proteins, nucleic acids, and lipids, thus resulting in the accumulation of oxidative damage in organisms [2].

The redox balance is physiologically regulated through the production and degradation of ROS in antioxidant systems to protect cells from oxidative damage. Superoxide dismutase (SOD) enzymes play a major role in the antioxidant system by catalyzing the conversion of superoxide radicals (O_2_^∙−^) to hydrogen peroxide (H_2_O_2_) and O_2_ [3]. In mammals, there are three SOD isoforms: CuZn-SOD (*Sod1*), which exists in the cytoplasm; Mn-SOD (*Sod2*), which is distributed in the mitochondrial matrix; and extracellular SOD (*Sod3*), which is localized in extracellular fluids, such as lymph, synovial fluid, and plasma.

Mice lacking* Sod2* showed dilated cardiomyopathy, steatosis, and metabolic acidosis, which resulted in neonatal lethality [4]. Therefore, heterozygous (*Sod2* ^+/−^) knockout or tissue-specific knockout mice are used to analyze the physiological role of* Sod2* in various tissues and organs [5, 6]. Carlsson et al. generated* Sod3*-null mutant mice [7]. Although* Sod3* ^−/−^ mice exhibited a shorter survival time than wild-type controls under hyperoxic conditions, the mice grew with no apparent abnormalities until late in life. In contrast, Reaume et al. first described the characterization of global* Sod1*-deficient (*Sod1* ^−/−^) mice. These mice exhibited marked vulnerability to motor neuron loss after axonal injury [8]. Subsequently,* Sod1* ^−/−^ mice showed a significantly shortened mean lifespan by approximately 30% and a high incidence of liver tumors by 20 months of age compared with those of* Sod1* ^+/+^ mice [9].* In vitro* studies also revealed that* Sod1* ^−/−^ fibroblasts showed a significantly decreased growth rate and higher sensitivity to O_2_ stress than* Sod1* ^+/+^ cells [10]. In the following paragraphs, we will introduce the various organ and tissue changes associated with the cellular phenotypes in* Sod1* ^−/−^ mice.

## 2. *Sod1* ^−/−^ Mice Exhibit Age-Related Pathological Changes in Various Organs and Tissues

We and other groups have demonstrated that* Sod1* ^−/−^ mice show various aging-like tissue changes, such as acceleration of Alzheimer's disease (AD) [11, 12], macular degeneration [13, 14], cataracts [15], dry eye [16, 17], cochlear hair cell loss [18], hearing loss [19], hemolytic anemia [20], osteopenia [21, 22], skin atrophy [23, 24], skeletal muscle atrophy [25], glucose intolerance [26, 27], hepatic carcinoma [9], fatty liver [28], infertility [29, 30], and luteal degeneration [31] (Table 1). Furthermore, the biochemical analyses revealed that* Sod1* loss in organs led to the accumulation of oxidative molecules such as carbonylated proteins, lipid peroxidants, oxidized nucleic acids, and advanced glycation end products (AGEs), which resulted in broadly impaired cellular signaling, gene expression, energy metabolism, cytoskeletal morphology, and cell death in the tissues.

## 3. Effects on the Individual Organs and Tissues

### 3.1. Effects on the Brain

Brain function declines in patients with neurodegenerative diseases, as well as during normal aging [32]. Ansari and Scheff reported a strong correlation between oxidative damage levels (total SOD, glutathione, catalase, thiobarbituric acid reactive substances, protein carbonyl, 3-nitrotyrosine, 4-hydroxynonenal, and acrolein) and the variable dementia status of subjects [33]. In addition, we have previously reported a specific reduction of SOD1 protein level, but not SOD2 and SOD3, in neocortex of AD brains [11]. We also reported that a mouse model for AD lacking* Sod1* showed exacerbation of memory loss and behavioral abnormalities associated with accelerated plaque formation and amyloid accumulation [11, 12]. Furthermore, a biochemical analysis also revealed high levels of intracellular N*ε*-(carboxymethyl) lysine (CML) and 8-hydroxy-2′-deoxyguanosine (8-OHdG) in the mouse brain. In addition,* Sod1* deficiency induced neuronal inflammation, as demonstrated by astrocyte and microglial activation in a mouse model for AD. These findings strongly suggested that SOD1 expression plays a pivotal role in maintaining cellular redox balance and brain function during aging.

### 3.2. Effects on the Eyes

Several eye diseases, such as age-related macular degeneration, cataracts, dry eye, phacoemulsification, and presbyopia, are closely related to the aging process [32, 34].* Sod1* deficiency induced the development of drusen-like deposits in the retina, choroidal neovascularization, and retinal pigment epithelium dysfunction, thus resulting in age-related retinal degenerative disorders, including age-related macular degeneration [13, 14]. An immunohistochemical analysis also revealed that CML-positive deposits were abundantly detected in the retinas of aged* Sod1* ^−/−^ mice [13]. Moreover, the* Sod1* ^−/−^ mouse lens showed twice the level of O_2_^∙−^ generation compared with that of control mice and had accelerated cataractogenesis following ultraviolet irradiation [15]. Furthermore, Dogru and colleagues reported that* Sod1* ^−/−^ mice also exhibited typical dry eye associated with lacrimal gland and meibomian gland changes, and this occurred in an age-dependent manner [16, 17]. The* Sod1* ^−/−^ lacrimal and meibomian glands showed increased 4-hydroxy-2-nonenal and 8-OHdG staining, apoptotic cells, and inflammatory infiltrates at 50 weeks of age compared to* Sod1* ^+/+^ mice. In addition, electron microscopy observations detected ultrastructural alterations in the mitochondria, including swelling, disorientation, shortening, disorganized cristae, marked fragmentation, shrinkage of the nuclei, and cytoplasmic vacuole formation, as well as the loss of nuclear membranes in* Sod1* ^−/−^ mice.

### 3.3. Changes in the Ears

The cochlear structure in the ear is progressively degenerated during aging, leading to hearing loss [35, 36]. McFadden et al. reported that* Sod1* deficiency morphologically induced a reduction of the inner and outer hair cells during aging [18]. In addition,* Sod1* ablation impacted the noise-induced permanent threshold shifts, leading to hearing loss [18, 19]. On the other hand, systemic overexpression of human* Sod1* protected against age-related and noise-induced hearing loss in C57BL/6 mice [37].

### 3.4. Changes in the Blood

During aging, the levels of oxidative stress markers, including 8-isoprostane and 2-thiobarbituric acid reactive substances (TBARS), are gradually increased in the plasma and erythrocytes of* Sod1* ^−/−^ mice [20]. Furthermore, Iuchi et al. reported that an intracellular ROS indicator, CM-H_2_DCFDA (DCF), in erythrocytes was spontaneously elevated in* Sod1* ^−/−^ mice.* Sod1* ^−/−^ mice also showed hemolytic anemia associated with splenomegaly. In fact, the erythrocyte lifespan from* Sod1* ^−/−^ mice was decreased by 60% compared to that of* Sod1* ^+/+^ erythrocytes [20]. We independently measured the serum levels of various markers of inflammation in* Sod1* ^−/−^ mice. A multiplex analysis revealed an altered pattern of inflammation markers, such as macrophage colony stimulating factor (M-CSF), macrophage inflammatory protein-1 beta (MIP-1 beta), macrophage inflammatory protein-1 gamma (MIP-1 gamma), regulated on activation, normal T cell expressed and secreted (RANTES), tumor necrosis factor-alpha (TNF-alpha), and thrombopoietin (TPO) in the* Sod1* ^−/−^ mouse sera (Table 2).

### 3.5. Effects on Bone

**Figure 1:**
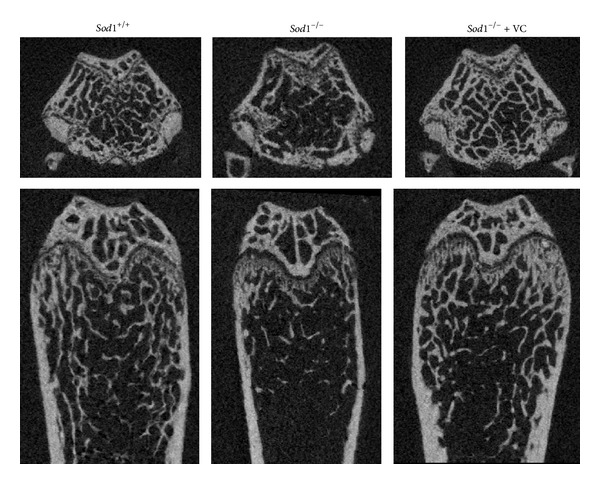
The bone loss in* Sod1* ^−/−^ mice. The treatment with 1% vitamin C in drinking water started from 4 weeks of age and continued for 12 weeks. Axial (upper panels) and coronal (lower panels) sections of *μ*CT images of the distal ends of the femora of* Sod1* ^+/+^ and* Sod1* ^−/−^ females at 16 weeks of age.

Aging stress generally causes bone loss and fragility [38]. We previously clarified that the loss of* Sod1* caused bone loss without leading to developmental skeletal abnormalities in both male and female mice [21]. The three-dimensional computed tomography analyses revealed that there was marked bone loss in both cortical and cancellous bones of* Sod1* ^−/−^ mice, which was associated with decreased bone formation and resorption, indicating the presence of low-turnover osteopenia (Figure 1).* Sod1* deficiency also enhanced the intracellular ROS production and the formation of pentosidine, one of the AGEs, in osteoblasts and bone [21]. Furthermore, Wang et al. also reported that young* Sod1* ^−/−^ mice showed bone fragility in the femora at the growth stage [39].

Recently, we found that mechanical unloading-induced bone loss associated with intracellular ROS generation in bone-forming cells and bone marrow cells [22]. Interestingly, we also detected specific* Sod1* upregulation at both the RNA and protein levels in bone during mechanical unloading [22]. Notably,* Sod1* deficiency significantly exacerbated the bone loss during mechanical unloading. In addition,* Sod1* ^−/−^ mice clearly displayed four-layered structural abnormalities and fragmented tidemarks in the enthesis, indicating tendon enthesis degeneration [40]. These findings suggested that* Sod1* plays a protective role in regulating bone and tendon enthesis homeostasis, as well as the redox balance during unloading and aging in mice.

### 3.6. Changes in the Skin

**Figure 2:**
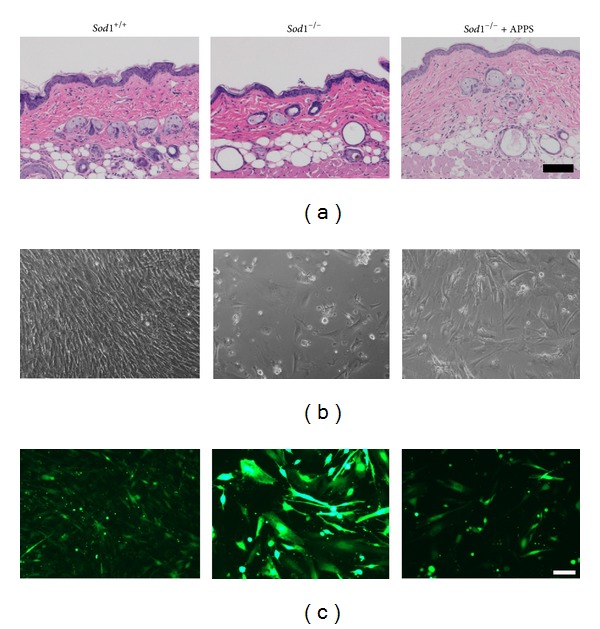
The skin and cellular phenotypes of* Sod1* ^−/−^ mice. (a) The hematoxylin and eosin staining of the back skin of* Sod1* ^−/−^ and* Sod1* ^+/+^ mice.* Sod1* ^−/−^ mice (5 months of age) were transdermally treated with 1% L-ascorbyl 2-phosphate 6-palmitate trisodium salt (APPS) for 4 weeks. (b) Dermal fibroblasts were dissected from* Sod1* ^−/−^ neonates at 5 days of age. The cells were cultured with or without 10 *μ*M APPS for 48 h under 20% O_2_. (c) The intracellular ROS levels in* Sod1* ^−/−^ fibroblasts treated with 10 *μ*M APPS were measured by examining the presence of CM-H_2_DCFDA. The scale bars represent 100 *μ*m.

Aged skin is characterized by wrinkles, sagging, dryness, and collagen degradation [41, 42]. We have previously reported that* Sod1* deletion caused typical age-related skin thinning [23]. In hematoxylin and eosin stained sections, the epidermis and dermis of the* Sod1* ^−/−^ back skin showed remarkable thinning (Figure 2(a)). In addition, the skin weight and hydroxyproline content, which is a unique amino acid present in collagen and elastin, in the* Sod1* ^−/−^ mice were compared with those of* Sod1* ^+/+^ mice [24]. An* in vitro* analysis using primary dermal fibroblasts from* Sod1* ^−/−^ neonates revealed severe cellular phenotypes, such as apoptosis and growth arrest, under normal conditions (Figure 2(b)). Furthermore,* Sod1* ^−/−^ fibroblasts showed excessive intracellular DCF-positive fluorescence (Figure 2(c)). Interestingly,* Sod1* ^−/−^ fibroblasts also had a significant enhancement of mitochondrial O_2_^∙−^ and impairment of the mitochondrial membrane potential [43].

### 3.7. Effects on Muscle

Aging contributes to the structural and functional changes in skeletal muscle in a wide range of mammals [44].* Sod1* ^−/−^ mice showed significant decreases in the whole hindlimb muscle mass compared with age-matched* Sod1* ^+/+^ mice, and this occurred in an age-dependent manner [25]. A biochemical analysis also revealed a significant increase in oxidative damage, such as the formation of F2-isoprostanes, protein carbonyls, and 8-OHdG, in* Sod1* ^−/−^ skeletal muscle [25].* Sod1* loss also induced aberrant mitochondria with abnormal shapes and led to lower ATP production in muscle. Mitochondria isolated from* Sod1* ^−/−^ muscle revealed significant increases in O_2_^∙−^ and H_2_O_2_ production and no compensatory upregulation of other antioxidant enzymes [45]. Recently, Zhang et al. reported that skeletal muscle-specific* Sod1* ^−/−^ mice failed to show muscle loss and ROS production [46]. Interestingly, a neuron-specific* Sod1* transgene in* Sod1* ^−/−^ mice prevented muscle loss [47]. The muscle from* Sod1* ^−/−^ mice with a brain-specific* Sod1* transgene did not show any differences in the muscle morphology, function, lipid peroxidation, or protein nitration compared with those of* Sod1* ^*+/+*^ muscle, suggesting that* Sod1* insufficiency in neuronal cells could lead to a dysregulation of the muscle mass and function in a nonautonomous manner.

### 3.8. Effects on the Pancreas

Aging stress also impairs insulin secretion and sensitivity in the pancreas [48]. Wang et al. reported that* Sod1* ^−/−^ islets exhibited a decreased *β*-cell mass, impaired glucose-stimulated insulin secretion, and a decreased ATP content, accompanied by elevated intracellular ROS levels [26]. In addition,* Sod1* ablation also downregulated the duodenal homeobox-1 (*Pdx1*) expression and forkhead box protein A2 (*Foxa2*) pathway in an O_2_^∙−^-dependent fashion by affecting these targets at the epigenetic, mRNA, and protein levels in the islets [26]. Furthermore, Muscogiuri et al. also showed that* Sod1* loss significantly impaired the glucose tolerance and led to a reduced *β*-cell mass, as well as insulin secretion in a hyperglycemic clamp test [27]. Interestingly,* Sod1* ablation failed to alter the peripheral and hepatic insulin sensitivity. These results proved that the absence of* Sod1* impaired *β*-cell function and glucose tolerance, but not insulin sensitivity, thus resulting in diabetes-like phenotypes.

### 3.9. Changes in the Liver

Aging of the liver is associated with an increased incidence of tumorigenesis [49]. The liver weight to body weight ratio in* Sod1* ^−/−^ mice was significantly higher than that of* Sod1* ^+/+^ mice [9].* Sod1* ^−/−^ mice also showed increased oxidative damage such as malondialdehyde (MDA), F2-isoprostane, and 8-OHdG accumulation in their livers [9]. In addition, the* Sod1* ^−/−^ livers showed an approximately 30% increase in hepatocarcinogenesis at 20 months of age compared to wild-type mice [9]. We also observed that* Sod1* ^−/−^ mice showed significantly accelerated hepatic lipid accumulation and peroxidation and impaired low-density lipoprotein secretion due to apoB degradation that occurred via a posttranslational mechanism [28]. Furthermore, Wang et al. reported that* Sod1* loss enhanced glycolysis and lipogenic signaling but decreased gluconeogenesis in the liver [50]. Recently, Kondo et al. described that the loss of senescence marker protein-30 (SMP30), which is a key enzyme required for L-ascorbic acid biosynthesis [51], accelerated the hepatic steatosis in* Sod1* ^−/−^ mice [52]. Both* Sod1* and* Smp30* deficiency led to a remarkable elevation of the triglyceride and cellular O_2_^∙−^ levels in the liver compared to those of* Sod1* ^−/−^ or* Smp30* ^−/−^ mice. These findings indicated that elevated oxidative stress and/or L-ascorbic acid depletion altered the glucose and lipid metabolism in the liver, suggesting that normal SOD1 expression is essential to maintain the hepatic glucose and lipid homeostasis.

In pharmacological studies, acetaminophen (APAP) injection induces glutathione depletion, the formation of reactive nitrogen species, and plasma ALT elevation, resulting in lethal hepatotoxicity in the case of an overdose [53]. Interestingly,* Sod1* deficiency attenuated the APAP-induced hepatotoxicity and lethality owing to its reduction of hepatic APAP-cysteine adducts, protein nitration, and CYP2E1 activity, which acts as an APAP-metabolizing enzyme, in the liver [53, 54]. These data indicated that the increases in intracellular O_2_^∙−^ caused by* Sod1* deletion inhibited CYP2E1 activity, thus resulting in protection against APAP-induced hepatotoxicity.

### 3.10. Effects on the Ovaries

Ovarian aging is characterized by a decline in the follicle numbers and sex steroid hormone secretion, which are associated with a gradual decline in fertility [55]. Although* Sod1* ^−/−^ female mice had normal estrous cycles and numbers of ovulated ova, their reproductive performance was inferior to that of female* Sod1* ^+/+^ and* Sod1* ^+/−^ mice [29, 30]. A hormonal analysis revealed that* Sod1* ^−/−^ females showed normal plasma levels of follicle-stimulating hormone (FSH), luteinizing hormone (LH), and estradiol at proestrus [31]. On the other hand, the plasma progesterone level was specifically repressed in* Sod1* ^−/−^ females compared to that in* Sod1* ^*+/+*^ females during pregnancy. Although* Sod1* loss in the ovaries and oocytes upregulated the intracellular ROS production,* Sod1* ^−/−^ oocytes could be normally fertilized and developed to the two-cell stage* in vitro* [31, 56]. However,* Sod1* ^−/−^ embryos failed to divide to the four-cell stage under conventional culture conditions (20% O_2_) [56]. When* Sod1* ^−/−^ embryos were cultured under hypoxic conditions (1% O_2_), they developed to the morula stage but could not develop into blastocysts [56], indicating that O_2_ stress inhibited the development of* Sod1* ^−/−^ embryos at the two-cell stage.

## 4. Intervention Strategies Using Antioxidants

Vitamin C (VC) is a soluble vitamin and the best characterized antioxidant [57]. In order to evaluate the protective effects of this antioxidant in* Sod1* ^−/−^ mice, we treated them with VC to try to rescue the organ phenotypes. Oral administration of VC suppressed the bone loss of* Sod1* ^−/−^ mice, indicating that O_2_^∙−^-induced bone loss could be improved by antioxidant treatment (Figure 1) [21]. In addition, VC treatment also normalized the bone strength and composition of collagen cross-links, without aberrant bone modeling [21]. We further applied a VC derivative, L-ascorbyl 2-phosphate 6-palmitate trisodium salt (APPS), on the* Sod1* ^−/−^ mouse skin. APPS is conjugated to a phosphate group and a long hydrophobic chain to promote stability and membrane permeability. The transdermal administration of the APPS reversed the skin atrophy and lipid peroxidation in* Sod1* ^−/−^ mice (Figure 2(a)).* In vitro* experiments revealed that APPS treatment completely improved the cell viability and suppressed the intracellular ROS production in* Sod1* ^−/−^ fibroblasts (Figures 2(b) and 2(c)). Furthermore, Iuchi et al. reported that oral N-acetyl cysteine (NAC) treatment attenuated the hemolytic anemia and inflammatory response, with ROS suppression, in the erythrocytes for* Sod1* ^−/−^ mice [20]. Additionally, we found that NAC treatment also improved the cell viability and decreased the intracellular ROS level in* Sod1* ^−/−^ fibroblasts [43].

The oxidative stress induced by* Sod1* deficiency is closely related to the progression of AD. Therefore, we hypothesized that antioxidant treatment would be able to alleviate the progression of AD. In this context, we treated mice with AD-like pathologies with VC. Confirming our hypothesis, chronic VC treatment restored the behavioral abnormalities, apparently by attenuating the oxidative stress in AD model mice [58]. VC also significantly suppressed the soluble A*β* accumulation in the brain, but not the plaque formation in the AD model mice [58]. Recently, we found that VC administration significantly prevented unloading-induced bone loss in wild-type mice [22]. These data strongly indicated that antioxidant intervention has remarkable protective effects against ROS-mediated tissue damage in mice.

## 5. Molecular Mechanisms Underlying the Organ and Tissue Pathologies in* Sod1* ^−/−^ Mice

To analyze the molecular mechanisms underlying the tissue damage induced by* Sod1* deficiency, we have investigated the phenotypes using double-knockout* Sod1* and liver-specific* Sod2* mice. As described above, the* Sod1* ^−/−^ mice showed acceleration of hepatic lipid accumulation, accompanied by increased oxidative damage. In contrast, liver-specific* Sod2* knockout mice did not show any obvious morphological abnormalities or spontaneous oxidative damage in the liver [59]. The double-knockout mice had an indistinguishable hepatic phenotype, including lipid peroxidation, lipid accumulation, and TG secretion, from that of* Sod1* ^−/−^ mice, indicating that the loss of* Sod2* failed to exacerbate the hepatic changes in* Sod1* ^−/−^ mice [28], demonstrating that the different enzymes do not have overlapping functions. Sentman et al. reported that combined* Sod1* and* Sod3* deficiency showed no additive effect on the lifespan and body weight in mice [60]. Likewise, Fujita et al. reported that* Sod1* and* Sod3 *double mutant mice showed the same phenotypes, such as O_2_^∙−^ and NO production and the TBARS level, in the kidneys compared with those of wild-type mice [61]. Moreover, glutathione peroxidase-1 (GPX1) loss also had no impact on the* Sod1* ^−/−^ phenotypes in the liver and pancreas [26, 62]. However,* Sod1* loss significantly decreased the GPX1 activity, but not the* Gpx1* level in the liver. The Lei group reported that* Sod1* loss increased the conversion of selenocysteine to dehydroalanine residues in the active site of hepatic GPX1, thus leading to proportional decreases in the activity of the enzyme as a whole [63]. Additionally, many reports have demonstrated that* Sod1* ^−/−^ mice showed no compensatory upregulation of antioxidant enzymes including* Sod2* and* Sod3* [43, 46, 60, 61]. These reports suggested that* Sod2*,* Sod3*, and/or* Gpx1* deficiency failed to further modify the organ pathologies in* Sod1* ^−/−^ mice.

Accumulating evidence suggests that both ataxia-telangiectasia mutated (ATM) and p53 play a central role in the DNA damage response induced by oxidative damage in organs and tissues [64]. In this context, Erker et al. investigated the organ phenotypes in mice lacking both* Sod1* and* Atm* to elucidate DNA damage response in the organs. The loss of* Atm* and* Sod1* did not show any interaction with regard to the overall cellular metabolism and survival in mice [65], indicating that* Sod1* regulates organ metabolism and lifespan in an* Atm*-independent manner.

Interestingly, we found that* Sod1* ^−/−^ skin displayed obvious p53 activation [43]. Additionally, treatment with a VC derivative remarkably suppressed the* p53* expression and oxidative damage in the skin of* Sod1* ^−/−^ mice, suggesting that the antioxidant activity of VC normalized the skin pathologies, at least in part, by suppressing O_2_^∙−^-mediated p53 activation* in vivo* [43]. Furthermore, the* Sod1* loss induced the phosphorylation of H2AX at Ser139 (*γ*H2AX), a DNA damage marker, and upregulated p21, a target gene of p53, in fibroblasts [43]. Of note, the* Sod1* ^−/−^ fibroblasts exhibited a loss of mitochondrial membrane potential and enhanced mitochondria ROS generation. Likewise, Muller et al. reported that* Sod1* ^−/−^ skeletal muscle showed significant alterations in mitochondrial function, including increased mitochondrial ROS generation and reduced ATP production [66]. Han et al. also revealed significantly higher levels of p53 and phospho-p53 in nuclei isolated from* Sod1* ^−/−^ livers [67]. Moreover, Wang et al. showed that* Sod1* ablation led to increased p53 and phospho-p53 levels in islets [26]. In humans, decreased* Sod1* expression and enhanced p53 expression were observed in AD-affected brain tissues [11, 68], osteoarthritic tissues [69, 70], bones in older individuals [71, 72], and tissues in infertility patients [73, 74]. Taken together, these data suggest that cytoplasmic SOD1 loss induced the DNA damage response, which was associated with p53 upregulation, resulting in age-related pathologies.

## 6. Conclusion and Perspective

In the present review, we introduced various organ and tissue phenotypes of* Sod1* ^−/−^ mice. Using* Sod1* ^−/−^ mice, we and other groups have demonstrated that* Sod1* deficiency enhances the intracellular O_2_^∙−^ production and oxidative damage, resulting in global, age-related pathological changes, including changes in the brain, eyes, ears, blood, bones, skin, muscles, pancreas, liver, and ovaries during aging. Antioxidant treatment prevented or improved the pathological changes in* Sod1* ^−/−^ organs and tissues. Interestingly,* Sod1* does not appear to interact with other major antioxidant enzymes, such as* Sod2*,* Sod3*, and* Gpx1,* in terms of the organ and tissues pathologies, as demonstrated using double-knockout mice. These lines of evidence strongly indicated that* Sod1* plays a central role in maintaining the cellular redox balance and organ function* in vivo*. We also suggest that p53 plays a fundamental role in* Sod1* ^−/−^-related pathologies. Further analyses will be needed to clarify the contribution of p53 to the molecular signaling and age-related pathological changes induced by* Sod1* deficiency, including those using double mutant mice with* Sod1* ^−/−^ and* p53* ^−/−^.

## Figures and Tables

**Table 1:** The age-related pathologies in *Sod1^−/−^* mice.

Brain	Acceleration of Alzheimer's disease	[11, 12]
Eye	Macular degeneration	[13, 14]
	Cataract	[15]
	Dry eye	[16, 17]
Ear	Cochlear hair cell loss	[18]
	Hearing loss	[19]
Blood	Hemolytic anemia	[20]
Bone	Osteopenia	[21, 22]
Skin	Skin atrophy	[23, 24]
Muscle	Skeletal muscle atrophy	[25]
Pancreas	Glucose intolerance	[26, 27]
Liver	Hepatocellular carcinoma	[9]
	Fatty deposits	[28]
Ovary	Infertility	[29, 30]
	Luteal degeneration	[31]

**Table 2:** The serum biomarker levels in *Sod1^−/−^* mice.

Markers	Concentrations	*Sod1^+/+^*	*Sod1^−/−^*	*P* value
IL-10	ng/mL	425 ± 54	451 ± 60	0.495
IL-11	pg/mL	39 ± 14.4	33 ± 13.2	0.627
IL-12p70	ng/mL	ND	ND	—
IL-17	pg/mL	ND	ND	—
IL-18	ng/mL	10 ± 1.1	12 ± 1.21	0.105
IL-1alpha	pg/mL	83 ± 63	134 ± 89.4	0.467
IL-1beta	ng/mL	12 ± 1.1	13 ± 1.6	0.268
IL-2	pg/mL	ND	ND	—
IL-3	pg/mL	ND	ND	—
IL-4	pg/mL	20.2 ± 0.0	20.2 ± 0.0	1
IL-5	ng/mL	0.23 ± 0.066	0.19 ± 0.055	0.406
IL-6	pg/mL	4.4 ± 1.7	18 ± 14.3	0.102
IL-7	ng/mL	0.02 ± 0.016	0.05 ± 0.025	0.296
IP-10	pg/mL	99 ± 17.1	150 ± 60.3	0.109
M-CSF	pg/mL	6.9 ± 0.50	6.0 ± 0.25	0.010∗
MCP-1	pg/mL	100 ± 34.2	124 ± 60.0	0.457
MCP-3	pg/mL	235 ± 50.9	235 ± 69.3	0.996
MCP-5	pg/mL	18 ± 1.7	24 ± 7.0	0.094
MIP-1alpha	ng/mL	1.6 ± 0.21	1.7 ± 0.09	0.307
MIP-1beta	pg/mL	55 ± 16.5	80 ± 17.5	0.005∗
MIP-1gamma	pg/mL	26 ± 3.6	34 ± 3.7	0.013∗
MIP-2	pg/mL	18 ± 2.1	20 ± 5.2	0.371
MIP-3beta	ng/mL	1.8 ± 0.37	1.7 ± 0.14	0.349
MDC	pg/mL	547 ± 234	626 ± 42	0.481
RANTES	pg/mL	0.26 ± 0.130	0.45 ± 0.049	0.014∗
TNF-alpha	ng/mL	0.066 ± 0.004	0.077 ± 0.010	0.041∗
TPO	ng/mL	75 ± 9.4	86.5 ± 5.6	0.049∗

ND indicates “not detected”. *indicates a significant difference.
